# Maternal Mortality in Rural South Ethiopia: Outcomes of Community-Based Birth Registration by Health Extension Workers

**DOI:** 10.1371/journal.pone.0119321

**Published:** 2015-03-23

**Authors:** Yaliso Yaya, Tadesse Data, Bernt Lindtjørn

**Affiliations:** 1 Centre for International Health, University of Bergen, Bergen, Norway; 2 Arba Minch College of Health Sciences, Arba Minch, Ethiopia; 3 Gamo Gofa Zone Health Department, Arba Minch, Ethiopia; Karolinska Institutet, SWEDEN

## Abstract

**Introduction:**

Rural communities in low-income countries lack vital registrations to track birth outcomes. We aimed to examine the feasibility of community-based birth registration and measure maternal mortality ratio (MMR) in rural south Ethiopia.

**Methods:**

In 2010, health extension workers (HEWs) registered births and maternal deaths among 421,639 people in three districts (Derashe, Bonke, and Arba Minch Zuria). One nurse-supervisor per district provided administrative and technical support to HEWs. The primary outcomes were the feasibility of registration of a high proportion of births and measuring MMR. The secondary outcome was the proportion of skilled birth attendance. We validated the completeness of the registry and the MMR by conducting a house-to-house survey in 15 randomly selected villages in Bonke.

**Results:**

We registered 10,987 births (81·4% of expected 13,492 births) with annual crude birth rate of 32 per 1,000 population. The validation study showed that, of 2,401 births occurred in the surveyed households within eight months of the initiation of the registry, 71·6% (1,718) were registered with similar MMRs (474 vs. 439) between the registered and unregistered births. Overall, we recorded 53 maternal deaths; MMR was 489 per 100,000 live births and 83% (44 of 53 maternal deaths) occurred at home. Ninety percent (9,863 births) were at home, 4% (430) at health posts, 2·5% (282) at health centres, and 3·5% (412) in hospitals. MMR increased if: the male partners were illiterate (609 vs. 346; p= 0·051) and the villages had no road access (946 vs. 410; p= 0·039). The validation helped to increase the registration coverage by 10% through feedback discussions.

**Conclusion:**

It is possible to obtain a high-coverage birth registration and measure MMR in rural communities where a functional system of community health workers exists. The MMR was high in rural south Ethiopia and most births and maternal deaths occurred at home.

## Introduction

The global maternal mortality ratios (MMRs) were halved between 1990 and 2010. However, of all maternal deaths in the world, 99% occur in low-income countries; 36 of the 40 countries with the highest MMR are in sub-Saharan Africa [1]. The MMR is the conventional key indicator to help monitor progress towards the MDG5 target of reducing maternal mortality by 75% in 2015 from the level in 1990 [2]. Unfortunately, measuring maternal mortality is difficult in low-income countries because of limited registration of births and deaths [3]. In 2013, UNICEF reported that 44% of births in sub-Saharan Africa, and only 7% in Ethiopia, were registered [4]. The continued failure of vital registration in low-income countries was noted as “*the single most critical development failure over the past 30 years*”[5].

Following the safe motherhood initiative (SMI) in 1987 and MDG declaration in 2000, several alternative approaches, such as survey methods [6–8] and statistical modelling of proxy data for national and international use [1,9], have been devised to estimate maternal mortality indices in low-income countries. While these methods provide important information for global and national planning, the evidence obtained using these techniques are often inconsistent and sometimes contradictory [10]. Progress towards the planned MDG goal and equity in health outcomes as well as post-MDG efforts require concrete data from population-based registries [11].

### The study in context

We conducted this study at a time when Ethiopia acknowledged the importance of and made practical movements towards improving maternal health and formulating a law for compulsory vital registration. As such, the five year (2010–2015) National Health Sector Strategic Plan emphasizes the intention to improve maternal and newborn health [12]. In addition, the government of Ethiopia was amidst discussions to pass a law for compulsory vital registration, which was approved in 2012 [13]. To implement the new registration law, the Central Statistical Agency (CSA) suggested the use of health extension workers (HEWs) as registrars of vital events in rural Ethiopia [14]. On the other hand, the World Health Organization (WHO) issued the maternal death review (MDR) and maternal death surveillance and response (MDSR) guidelines as part of international efforts to reduce maternal mortality [15,16]. When we conducted the current study, however, there was a limited such committees or systems for reviewing maternal deaths in rural Ethiopia. As a result, we did not know whether the continuous registration of births and surveillance of maternal deaths could result in intended outcomes of complete birth registration and maternal mortality measurement in rural areas of Ethiopia.

In 2003, Ethiopia adopted a community-based health extension programme (HEP) [17], and currently over 38,000 HEWs are working in all rural villages. However, the opportunity for using these community health workers to obtain useful data for public health policy and action has not been explored. Consequently, the objective of this study was to assess whether HEWs can effectively register births and actively identify maternal deaths in the rural villages to measure the magnitude and associated factors for maternal mortality through a community-based birth registration system.

## Methods

### Ethics statement

The Ethical Review Committee for the Health Research of Southern Nations Nationalities and Peoples’ Regional State (SNNPRS) Health Bureau in Ethiopia, and the Regional Committee for Health Research Ethics of North Norway (REK Nord) in Norway approved the study. Birth and birth-outcome registration is part of the routine work of the HEWs in Ethiopia, which is acknowledged by the government. We systematized the registry by preparing a standardized format and providing technical support. Personal identifiers were removed from the stored data used for research. We obtained informed verbal consent from respondents for the validation study of house-to-house survey and the responses were recorded on the questionnaire as “accepted” or “declined” to participate. Written consent was not considered because a large number of the respondents were illiterate and the Ethics Committee approved the verbal consent procedure.

### Study area

**Fig 1:**
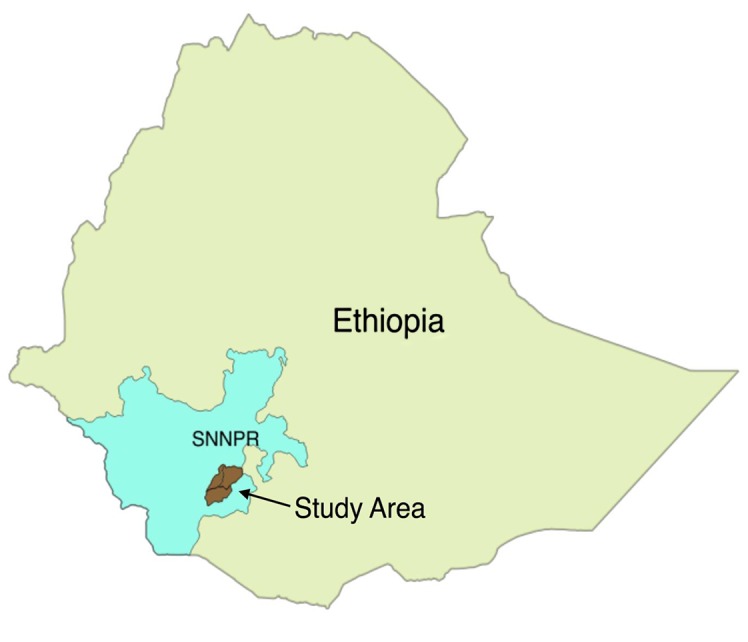
The map of the study area within southern Ethiopia.

The Ethiopian government has autonomous regional states within the Federal Republic. In turn, regional states are subdivided into zones (provinces), Woredas (districts), and Kebeles (villages). A zone is a cluster of 10–15 districts, and a district is a group of 20–50 villages. A Kebele is the lowest administrative structure and is comprised of 1,000–1,500 households. This study was conducted in three districts (Arba Minch Zuria, Bonke, and Derashe) in two zones (Gamo Gofa and Segen Area Peoples') in the Southern Nations, Nationalities, and Peoples’ Region (SNNPR, Fig. 1). The Gamo Gofa Zone (population = 1,740,828 people in 2010) [18], the centre of which is at Arba Minch, is 505 km from Addis Ababa to the southwest and the Segen Area Peoples’ Zone (636,794 residents in 2010) [18] is 575 km from Addis Ababa.

Bonke, with a population of 166,913 people in 2010, had no hospital providing comprehensive emergency obstetric care at the time of the study. The nearest such service was at Arba Minch Hospital, which is 50–150 km from the villages of Bonke. Arba Minch Zuria, with a population of 179,785 people, has a hospital, although the largest proportion of the population lives in the highlands far from the hospital and driveable roads. Derashe, with a population of 141,589 has a district hospital in the main town of Gidole, as well as well-functioning maternity waiting homes, traditional thatched huts built in the hospital compound, where mothers with high-risk pregnancies are referred and observed until delivery [19].

### Sampling and study participants

**Fig 2:**
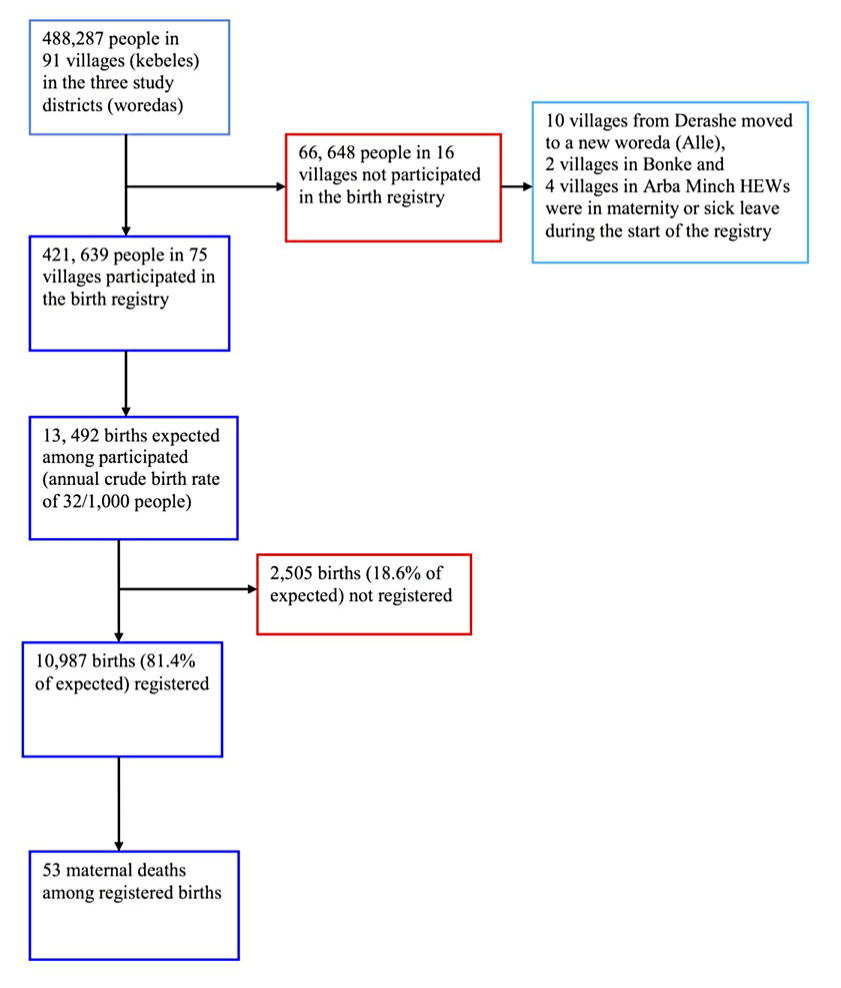
The profile of the birth registry.

Fig. 2 presents the study profile. In 2008, the MMR in Ethiopia was 590 per 100,000 live births (LBs) [9]. Assuming this would be comparable for the study area, we expected there could be a 10% decline in two years resulting in an MMR of 531 (95% CI: 413, 669) per 100,000 LBs in 2010. Thus, we expected 70 maternal deaths in a year (95% CI: 55, 88) out of estimated 13,492 births (13,223 LBs) in a population of 421,639 people. LBs were approximated 98% of all births in the area [20]. To estimate the expected number of births, we used an annual crude birth rate (CBR) of 32 per 1,000 population based on the following two sources of birth rate information: a finding from a household survey in 2010 in one of the study districts (Bonke) [20], and the same estimate by The World Bank of CBR in Ethiopia for 2010 [21]. To identify group differences in the MMR, we assumed the number of maternal deaths amongst births determined above would provide sufficient data.

We purposely selected three districts with the number of residents expected to produce the above estimated births and maternal outcomes. The districts were assumed to represent the area in terms of health services, demographics, and road access. In these districts, we included all kebeles (villages), except those where the HEWs were sick or on maternity leave at the time of starting the registration. We used OpenEpi software (Open Source Epidemiologic Statistics for Public Health version 3.01,www.openepi.com) to calculate the sample size.

### The HEP and the HEWs

The HEP is a community-based healthcare system with two female HEWs serving a rural village of 1,000–1,500 households. Most of the HEWs have completed a 10^th^ grade education and received one year of general health training. Their work focuses on family health (child vaccinations, family planning, antenatal care, and assisting normal deliveries) and health promotion. HEWs are expected to routinely visit each household in their catchment once a month, prioritizing households with pregnancies, newborns, and sick persons. HEWs are part of the permanent health workforce and receive a monthly salary of 40–50 USD from the government based on their years of service. In addition, 5–10 lay-women known as volunteer health promoters (VHPs), assist the work of HEWs by informing of households with a recent delivery, sick people, and deaths in the sub-villages.

### Data collection procedures (the birth registry)

We conducted one week training at each woreda centre for HEWs, supervisors, and the district health authorities before the registry started. Supervisors were experienced nurses (one per district), who helped the HEWs in reviewing and classifying deaths, monitoring the quality of data, and transferring the registered information from HEWs to the central data clerk. During the training, we clarified the WHO ICD-10 definition and classification of maternal deaths [22]. Accordingly, if a woman died during ante- or intra-partum periods, or within six weeks after termination of a pregnancy and her pregnancy status was known, her death was considered a maternal death if the death was not because of an accident or incident such as suicide. We also used extractions from the WHO maternal death review (MDR) manual published in 2004 to determine the cause of deaths [15]. As such, diagnosing the cause of death was based on symptomatic approaches such as convulsions attributed to hypertensive disorders, fevers to infections, and excessive bleeding due to haemorrhage.

The specific registration and maternal death ascertainment procedure is presented as follows. HEWs visited homes within hours or days after the pregnancy ended depending on the distance and the speed of notification from the sub-village VHPs or families. At the household, HEWs assessed and registered birth and births conditions. The HEWs continued the follow-up until a maternal death was occurred or six-week post-partum. This collection of information was similar to births that occurred at home and in health facilities because all births were available for recording at homes. In addition, in households in which a woman of reproductive age died without giving birth, HEWs critically reviewed the conditions at the time of death to determine the pregnancy status of the deceased and determine the probable cause of death. Husbands or fathers of the baby (FOBs) were primary sources of information for maternal deaths; however, in the cases where obtaining information from the husbands or FOBs was not possible, adult members of the family helped in providing information.

**Fig 3:**
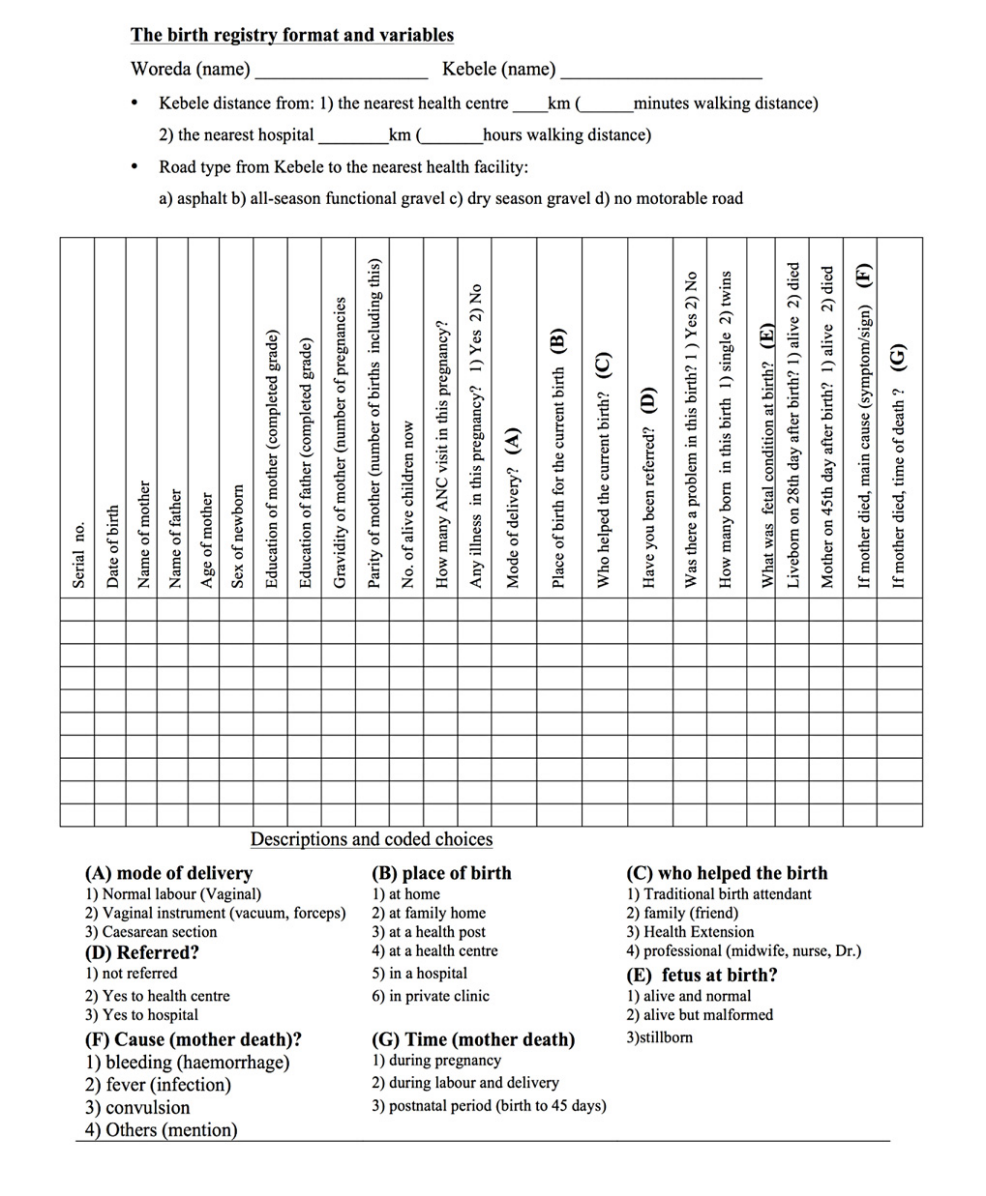
The birth registry format.

HEWs registered the data in printed birth registry books (Fig. 3). The book contained important socio-demographic variables, such as the distance of the village from the nearest health centre and the nearest hospital recognized by the respective district health offices, as well as the type (quality) of road to the village as a general heading information. The actual body of the book rows contained personal background information, such as education of the mother and father and age of the mother. In addition, the woman’s parity, the place of birth, the attendant of birth, the condition of the newborn at birth (alive or stillbirth), the gender of the foetus, and maternal deaths (including the place, cause, and time) were among the variables. Registration was made in duplicate and the first copy was detached and sent to the Research and Training Centre at Arba Minch Hospital, while the second copy remained with the book in the village. Most births were registered within 24 hours of delivery, unless there was a special reason for a delay (births in distant health institutions, where the household was far from the HEW station or HEWs were not informed in a timely manner). Similarly, most maternal deaths were identified immediately. Nevertheless, HEWs made a final follow-up home visit six weeks after birth or abortion when death information was not obtained prior to the stated deadline.

### Outcomes

The primary outcomes were the coverage of birth registration (percentage registered out of the estimated) and the MMR. The secondary outcome was the proportion of skilled birth attendance, facility deliveries supervised by skilled professionals.

### Data quality control (the validation study)

To check the validity of the registration eight months after the start of the registration, we conducted a house-to-house survey in 15 of the 30 rural villages in the Bonke. Data collectors who had completed the 12^th^ grade visited every household and searched for a birth or pregnancy outcome since the start of the birth registry. For births already registered in the birth registry, they checked the content (date of birth, date of death, and baby’s gender). The unregistered were recorded and the data were transferred to the registry book. Based on the findings of the validation study, we discussed the feedback with the HEWs and supervisors to improve the coverage of the registration.

### Data analysis

We entered, checked, and analyzed the registry and validation data using the statistical package for social sciences (SPSS-16) describing the results in tables showing proportions and means. To show the variation in maternal mortality, we used a chi-square test. For the validation study, we produced a descriptive table showing the proportion of births registered and unregistered out of the births found during the validation survey. We made a cross-tabulation for crude analysis to determine the risk of maternal deaths among registered births compared to unregistered and the effect of antenatal follow-up and distance from HEW station on the likelihood of births being registered.

## Results

### Socio-demographic characteristics of registered births

Table 1 presents the background information about the parents and the maternal services received during pregnancy and delivery. We registered 10,987 births (5,612 [([51·1%] boys and 5,375 [48·9%] girls). The average age of the mothers was 28·1 (SD = 4·5) years, and the median number of pregnancies was 3 (IQR = 2–5). The illiteracy rate was 77% (8,454/10,987) among mothers, and 54·6% among the husbands and FOBs (6,001/10,987). The median distance to health centres was 10 km (IQR = 5–18), and 57% (6,236/10,987) of the births had a health centre within 10 km. The median distance to hospitals was 40 km (IQR = 24–67).

**Table 1:** Socio-demographic data on parents, services, and infrastructures in the birth registry districts of south Ethiopia in 2010.

Background variable	Value
Age of mother (years): mean (SD)	28.1 (4·5)
**Education of fathers N = 10,987** *	
Illiterate: no. (%)	6,001 (54·6)
Literate: no. (%)	4,986 (45·4)
**Education of mothers N = 10,987** *	
Illiterate: no. (%)	8,454 (77·0)
Literate: no. (%)	2,533 (23·0)
Health Centre distance (km): median** (IQR)	10 (5–18)
Hospital distance (km): median (IQR)	40 (24–67)
Pregnancy (gravidity): mean (SD)	3.7 (2·2)
**Antenatal visits: mean (SD) N = 10,987**	2.4 (1·4)
No antenatal: no. (%)	1,655 (15·1)
1–2 visits: no. (%)	3,767 (34·3)
3 or more: no. (%)	5,565 (50·7)
**Place of Delivery (%): N = 10,987**	
Home: no. (%)	9,863 (90)
Health post: no. (%)	430 (4.0)
Health Centre: no. (%)	282 (2·5)
Hospital: no. (%)	412 (3·5)
**Road access (%): N = 10,987**	
All-weather: no. (%)	4,214 (38·4)
Dry weather: no. (%)	5,446 (49·6)
No car road: no. (%)	1,327 (12·0)

Note:

* Education: illiterate are those cannot read/write and had no formal education, literate include those who can read/write and completed higher education,

** 57% of households with births were within 10 km of health centres (10 km is the government target for access)

### Completeness of birth registration

The 10,987 registered births were 81.4% of the 13,492 expected births based on the annual CBR for the year. Because the CBR was estimate-based, we made sensitivity analysis of the expected births by increasing the annual CBR by two to 34 and again by decreasing by two to 30 per 1000 population. If the CBR was 34, the number of expected births was 14, 336 of the 421, 639 residents in the registry villages. This decreased the registration coverage from 81.4% to 77%, and if the CBR was 30, the registration coverage rose to 87% from our best estimate of 81.4% because the expected number of births was 12,649.

### Maternal mortality outcomes

We registered 53 maternal deaths (Table 2), yielding an MMR of 489 per 100,000 live births The MMR of 489 per 100,000 live births was within the 95% confidence interval of the expected MMR of 531 (95% CI: 413–669). Table 3 compares the MMR estimates between the birth registry (489), the validation study (474), the previous household survey finding from the area (425), and the national estimates by IMHE (590), UN (350), and DHS (676) per 100,000 live births. In the birth registry study, five mothers (9·4%) died during pregnancy, 21 (39·6%) died during labour and 27 (51%) died within six weeks post-partum. Of the maternal deaths, 35.8% (19/53) were due to bleeding, 22·6% (12 /53) due to infection, 17% (9/53) because of hypertensive disorders, 13·2% (7/53) because of obstructed labour, and 11% (6/53) registered as “others”. Additional inquiries indicated that three of the six cases categorized as ‘other causes’ and two cases in the haemorrhage category were probable complications of abortion. Of all 53 maternal deaths identified, 83% (44) occurred at home and 17% (9) were in health institutions.

**Table 2:** District distribution of births, birth outcomes, and services during pregnancy and delivery in south Ethiopia in 2010.

	Districts			
	Derashe	Bonke	Arba Minch	Overall
All births	3,134	3,571	4,282	10,987
Live births	3,099	3,529	4,205	10,833
Maternal deaths (number)	14	18	21	53
**Antenatal visits: Mean (SD**)*	2·5 (1·6)	2·1 (1·4)	2·5 (1·3)	2·4 (1·4)
No antenatal: no (%)	546 (17·4)	669 (18·7)	440 (10·3)	1,655 (15·1)
1–2 visits: no (%)	826 (26·4)	1,418 (39·7)	1,523 (35·6)	3,767 (34·3)
3 and more: no (%)	1,762 (56·2)	1,484 (41·6)	2,319 (54·1)	5,565 (50·6)
**Place of delivery no (%) N = 10,987**				
Home	2843 (90·7)	3,183 (89·1)	3,837 (89·6)	9,863 (90)
Health post	40 (1·3)	290 (8·2)	100 (2·4)	430 (4·0)
Health Centre	25 (0·8)	81 (2·2)	176 (4·1)	282 (2·5)
Hospital	226 (7·2)	17 (0·5)	169 (3·9)	412 (3·5)
**Delivery attendant no (%)**				
Family member or relative	234 (7·5)	1,823 (51·1)	1,937 (45·3)	3994 (36·4)
Traditional birth attendant	2,434 (77·7)	1,027 (28·8)	1,364 (31·8)	4825 (44·0)
Health Extension Worker	215 (6·8)	623 (17·4)	636 (14·9)	1,474 (13·4)
Health professionals (skilled)	251 (8.0)	98 (2.7)	345 (8.0)	694 (6.3)

Note:

* SD, standard deviation

### Place of birth and assistance during labour

Table 2 also shows the place of delivery and who assisted the births. Of the registered births, 85% of the mothers (9332/10987) received antenatal care. However, 90% of the mothers (9863/10987) delivered at home, 4% (430) at health stations (two-room buildings staffed by HEWs), 2·5% (282) at health centres (staffed by nurses and midwives), and 3·5% (412) in hospitals. One-third of the births were (36·4% [3,994/10,987]) were assisted by family members and relatives, 44% (4,825) by traditional birth attendants (TBAs), 13·4% (1,474) by HEWs, and 6·3% (694) by skilled professionals (physicians, health officers, midwives, and nurses). Regarding road access, 61·6% of the births (6,773/10,987) took place in villages without driveable-road or access to driveable road during the dry season only.

### Variations in maternal mortality

The MMR was similar in the three districts, and there was no difference between educated and illiterate mothers (Table 3). However, the MMR was higher in the villages where there was no driveable road access compared to villages where there was at least a dry-weather road (946 vs. 446) and an all-weather road [946 vs. 410; χ^2^ (df): 6·11 (2), p = 0·039]. Maternal mortality was six times higher among mothers who had illness of any kind during pregnancy, compared to those who had not complained of any illness [1,763 vs. 306; χ^2^ (df): 48·6 (1), p < 0·0001], and increased when the male partners were illiterate, compared to those who were literate [609 vs. 346; χ^2^ (df: 3·8 (1), p = 0·051].

**Table 3:** Variations in maternal mortality across variables, south Ethiopia, 2010.

Variable	Maternal deaths	Live births	MMR*(95% CI)	p-value (2-tail)
**District**				
Bonke	18	3,529	510 (312, 789)	
Arba Minch	21	4,205	500 (318, 750)	
Derashe	14	3,099	452 (257, 740)	
**Maternal age**				
≤ 20	4	709	564 (180, 1,355)	
21–35	45	9,475	475 (351, 630)	
≥ 36	4	649	616 (196, 1,480)	
**Parity (no. of births)**				
≤3	32	5,760	556 (386, 774)	
≥4	21	5,073	414 (263, 621)	
**Mother’s education** **				
Illiterate	40	8,331	480 (347, 647)	
Literate	13	2,502	520 (290, 865)	
**Father’s education** **				
Illiterate	36	5,913	609 (433, 833)	0·051
Literate	17	4,920	346 (208, 541)	
**Antenatal visits**				
No ANC	11	1,628	676 (356, 1,172)	
1–2 visits	17	3,711	458 (276, 717)	
3 or more	25	5,494	455 (301, 661)	
**Place of birth**				
Home	44	9,757	451 (332, 599)	
Institution	9	1,066	844 (412, 1,544)	
**Distance to health centre**				
≤10	35	6,151	569 (403, 782)	
≥11	18	4,649	387 (237, 600)	
**Distance to hospital**				
≤25	14	3196	438 (250, 716)	
≥26	39	7,604	513 (370, 693)	
**Road to the village** ^§^				
All weather drive	17	4,150	410 (247, 642)	
Dry weather drive	24	5,377	446 (293, 653)	
No driveable road	12	1,269	946 (513, 1,602)	0·039
**Sickness in pregnancy**				
Yes	24	1,361	1,763(1,159, 2,572)	0·000
No	29	9,472	306 (209, 433)	
Total	53	10,833	489 (366, 628)	

Note:

*maternal mortality ratio per 100,000 live births. Cells with P-value > 0.05 (with no statistical significance) are left empty. MMR in the parenthesis are 95% CIs.

**Education: illiterate are those who cannot read/write and had no formal education, literate include those who can read/write and more educated up to higher education.

^§^ Compared only all-weather road against no driveable road

### Results from the validation study

Table 4 shows findings from a house-to-house validity study compared to the birth registry. Of the 2,401 births identified during house-to-house checks, 1,718 (71·6%) were registered and 683 (28·4%) were not registered. Births to women who attended antenatal clinics (ANCs) were more likely to be registered compared to mothers who did not have any ANC visits (74·6% [1413/1895] vs. 60·3% [305/506]; RR = 1·24 [95% CI: 1·15–1·33]). Births that had occurred within 5 km from a HEW station were also more likely to be registered compared to births that occurred > 6 km from a HEW station (75·2% [1072/1425] vs. 66·2% [646/976]); RR = 1·14 [95% CI = 1·08–1·20]). The MMR was similar among registered and unregistered (474 vs. 439) per 100,000 LBs, (RR = 1·06 [95% CI: 0·28–3·98]).

**Table 4:** Results from house-to-house validation study to check completeness of birth registry in south Ethiopia during 2010.

Variables	registered number (%)	unregistered number (%)	checked (100%)	RR** (95% CI)
All births	1,718 (71·6)	683 (28·4)	2,401	
Live births	1,698 (71·5)	675 (28·5)	2,373	
Maternal death	8	3	11	
MMR*	474	439	464	1.06 (0.28, 3.98)^§^
Antenatal care visit				
Yes	1,413 (74·6)	482 (25·4)	1,895	1.24 (1.15, 1.33)
No	305 (60·3)	201 (39·7)	506	Ref
Distance to HEW^β^ station				
≤ 5 km	1,072 (75·2)	353 (24·8)	1,425	1.14 (1.08, 1.20)
≥6 km	646 (66·2)	330 (33·8)	976	Ref

Note:

* MMR, maternal mortality ratio per 100,000 live births,

** RR, relative risk,

§ relative risk of MMR among registered births compared to unregistered. HEW^β^, health extension worker

### Comparing maternal mortality in the study area with national estimates

To provide a better understanding of the MMR in the study area obtained by using different methods of measurement with the national estimates for Ethiopia, we provide a summary table (Table 5).

**Table 5:** Estimates of the maternal mortality ratio per 100,000 live births in the study area compared with national estimates in Ethiopia during the years around 2010.

MMR^β^	Year	Source	Estimated for	Remarks
489	2010	Birth registry	Study area	This paper
474	2010	Validation of birth registry	study area	This paper
425	2010	Household survey	Study area	Yaya et al [20]
350	2010	UN modelled estimate	National level	WHO and co [1]
676	2010	DHS*	National level	CSA-Ethiopia** [6]
590	2008	IHME modelled estimate***	National level	Hogan et al [9]

Note: MMR,

^β^ maternal mortality ratio per 100,000 live births, DHS,

* Demographic and Health Survey, CSA,

** Central Statistical Agency IHME,

*** Institute of Health Metrics and Evaluation, Washington University, USA.

## Discussion

In 75 rural kebeles, we registered 81% of expected births in nearly half a million residents. The MMR was 489 per 100,000 LBs. In addition, four of every five maternal deaths occurred at home without the attention of health facilities in the area. A validation study also showed a high initial coverage of birth registration out of births identified through checks by household visits, and there was a similar MMR between registered and unregistered births. These findings suggest that it is possible to register a high percentage of births expected in rural communities using community health workers. In addition, the community-based birth registry appears to be a useful tool to identify and measure maternal mortality. Majority (90%) of the registered births took place at home while > 90% of maternal deaths occurred during intra- and post-partum periods. Haemorrhage and infections were the leading causes of maternal deaths. The MMR was higher in remote areas without roads, amongst couples in which the male partners were illiterate, and among mothers who experienced illnesses during pregnancy.

We are not aware of other studies that have tested the feasibility of registration of births and pregnancy outcomes such as maternal deaths in rural Ethiopia. The 2013 UNICEF report showed that only 7% of births are registered in Ethiopia, which is the third lowest in the world ahead of Liberia (4%) and Somalia (3%) [4].

Unfortunately, because of resource constraints we were not able to conduct a baseline survey of fertility for 2010 in the study area. Consequently, we assessed the completeness of the birth registry using the coverage of the registry out of expected (estimated) births in the area, depending on a finding from a previous survey (CBR = 32 per 1,000 population between 2006 and 2010) [20]. Expecting the number of births through the annual CBR estimation may under- or over-estimate the true number of births, and the coverage may vary from the 81.4% we report. Additionally, we conducted a validation study for births occurring in 8 months, and this presents a problem in measuring the annual CBR. As such, the validation study only serves the purpose of determining what proportion of actually observed births at households were registered and whether or not there was a difference in an outcome of interest (MMR) between registered and unregistered births. The validation study cannot help to estimate annual CBR that needs 12 months of data on births.

Given the continuous decline in fertility in rural areas, as indicated by a decrease in CBR per 1,000 population (43 in 2000 to 28 in 2014) according to the DHS showed by findings from DHS [23], the CBR in 2010 may have been < 32 per 1,000 people or we are uncertain whether or not the CBR was even higher. We did a sensitivity analysis by using annual CBRs of 34 and 30 to compare the registration coverage against the CBR of 32 used in the analysis. The analysis provided registration coverage between 77% and 87%. The true CBR in the area may be between 30 and 32 per 1,000 population, which implies that the actual coverage of birth registration may have been > 81%. Therefore, we argue that the registration coverage for the study was high for a beginning community-based birth registry in rural settings.

Nevertheless, the uncertainty on the number of expected births does not affect our MMR because we calculated MMR from registered births rather than expected births. The concern, however, is whether there was a difference in MMR between registered and unregistered births. We attempted to address this partly by doing a validation study, which resulted in a similar MMR between the two groups. Theoretically, both the continuous birth registration and the validation survey may give an under- or over-estimate of the MMR. In practice, the problem related to measuring maternal mortality is under-reporting rather than over-reporting. As such, reported maternal deaths under estimate up to 30% of the actual MMR worldwide [16].

Important factors related to under-reporting of maternal mortality among registered pregnancy outcomes are problems of identifying early pregnancy maternal deaths before the pregnancy is clearly recognized and deaths due to abortion because of secrecy and stigma [24]. Thus, we made a rigorous effort to review every adult woman’s death to determine whether it was pregnancy-related, mainly by using the advantage of a close relationship between HEWs and the people in their villages. Nevertheless, we recognize that it is difficult to avoid under-reporting and we cannot estimate how many early pregnancy, and abortion-related deaths went unnoticed. Future studies may consider beginning the registration of pregnancies (instead of pregnancy outcomes used in this study) to better capture maternal deaths during pregnancy. Another important concern of under-reporting is the limited knowledge of the proportion of maternal deaths among unregistered pregnancy outcomes. Our validity check showed no difference in the MMR between registered and unregistered births. Nevertheless, because the validation study took place in 15 of 75 villages during the 8^th^ of a 12-month registration, there may have been limitations to representing a complete picture of similarity.

While these limitations are acknowledged, we have reported an MMR estimate closer to reality and the MMR herein may be one important step in measuring maternal mortality through a community-based birth registry in rural Ethiopia. Furthermore, the current finding of the proportion of maternal deaths at facilities and homes is also consistent with our previous report [20,25]. In addition, our findings showed that 83% of maternal deaths occurred at home without access to a health institution, which is similar with the results of a study from Mozambique that tracked maternal mortality through active community-based approaches, and showed that health institutions did not identify 86% of maternal deaths that occurred in their area [26]. The percentage of maternal deaths at home was less than the proportion of births in the same place (83% vs. 90%). The reason could be that some severe cases selectively visited health facilities and died in the facilities. The severity-based selective facility utilization argument is also supported by the findings of a higher MMR among facility births compared to births at home (844 vs. 451) in the current study. These results have two implications. First, the finding of four of five maternal deaths occurring at home means health facilities had no means to avoid these deaths and were not able to identify and record when the deaths occurred. Second, the higher MMR among facility deliveries implies that the health institutions had low ability to save mothers who had sought help.

In the study area, we have limited documentation to describe progress in terms of maternal mortality in previous years. A household survey in one of the districts to measure maternal mortality between 2006 and 2010 provided an MMR of 425 per 100,000 LBs [20], which is similar to the current finding. In addition, we published maternal mortality indices (MMR and lifetime risk) from the area using the indirect sisterhood method [27]. Nonetheless, because of the indirect nature, the estimate refers to a time of more than a decade ago and indirect sisterhood estimates provide the order of magnitude with limited value to oversee trends.

The MMR we report (489 per 100,000 LBs in 2010) is higher than the level of reduction required to attain the MDG5 target (from 968 in 1990 to 242 in 2015 in Ethiopia) [9]. The MMR was also higher than the joint estimate provided by WHO, UNFPA, UNICEF, and The Wold Bank (350) for 2010 in Ethiopia [1], but lower than the DHS reported national estimate (676) for a similar period [28]. However, our finding was similar to most results from previous community-based studies in Ethiopia. The MMR per 100,000 LBs was 402 in Jimma (1990) using a cross-sectional survey [29], 440 in Butajira (1996) using a surveillance approach [30], 570 in Illubabor (1991) using the indirect sisterhood method [31], and 566 in Addis Ababa (1983) using a household survey [32].

Nevertheless, given decades of time between the aforementioned studies and the current study, the MMR may have been different in this study area compared to those provinces. An alternative explanation could be that the survey and periodic surveillance techniques used in the aforementioned studies resulted in under-estimations of MMR at that time. This could also be explained by the fact that Ethiopia had a high MMR estimate nationally during the time when these studies were conducted [33]. As such, the prospective method we used and the presence of the HEWs within the villages that increases the awareness of important events might have helped our study provide a better estimation of the current MMR.

We showed that there was no difference in the MMR between districts with better health institutions (Arba Minch and Derashe) and a district with less poor health facilities (Bonke). This may be explained by the low utilization rate of existing health institutions for deliveries in all the districts. Hence, this emphasizes the importance of improving people's behaviours with respect to utilizing institutional delivery by skilled professionals in addition to distributing and strengthening health facilities. The findings of a high MMR in the remotest villages without driveable roads highlight the inequalities in health outcomes experienced by these women. Maternal mortality was also higher where husbands and FOBs were illiterate. This may explain the importance of male partners as decision makers for receiving care (health), as well as their general contribution as providers to improved living conditions (wealth). This finding is in agreement with the suggestions of the Oxford Multi-dimensional Poverty Index (MPI) Study, which described the importance of an educated person in a household for a positive health outcome [34]. In addition to the aimed outcomes, the study also showed the existence of substantial missed opportunities in maternal care by the health system. Fortunately, 85% of pregnant women visited health workers for antenatal check-ups at least once, whereas only 10% returned for delivery at the health facilities for supervised delivery.

The weaknesses of the current study follow. First, although we made a rigorous effort to identify early maternal deaths, an important limitation of the study was our inability to explain the amount of unnoticed early pregnancy-related maternal deaths. Specifically, we might have missed some of the abortion-related deaths because of the stigma and secrecy associated with abortion. Second, although we achieved a high coverage of > 80% registration, not all expected births were registered, thus leaving concerns about whether or not the MMR is higher or at least different among unregistered births. Our finding was a significant increase from the 7% national birth registration in Ethiopia [4], and almost non-existent in rural areas. Third, despite similar MMRs between registered and non-registered births, our validation study showed that HEWs were more likely to register births that occurred near their station and those who attended antenatal care, highlighting potential selection bias. Fourth, the death ascertainment method through the HEWs was less valid compared to the standard techniques of death confirmation by physicians or using autopsy [35].

Nevertheless, we believe that the diagnostic technique we used is better than asking a family member about a death that occurred years ago in survey studies. Furthermore, the standard methods of ascertaining maternal death are less likely to reach rural communities in low-income countries. Hence, it may be important to use and improve available opportunities, such as community health workers (HEWs in Ethiopia) to prospectively identify, review, and record maternal deaths, even when it means less accurate diagnosis of the cause of death in resource-limited settings.

Finally, because of the short period of observation, we cannot show in this paper whether community-based registration contributes for the reduction of MMR. Future studies may try to address these concerns. In addition, although it is difficult to suggest similar achievements in all areas, the study can be repeated in any part of Ethiopia because all villages have the same HEWs working under similar conditions. The same can also be applied in low-income countries that have an organized community health workforce.

## Conclusion

It is possible to register most births in rural Ethiopia through the HEWs and use the registry as a tool to measure maternal mortality. The MMR was high in the study area compared to the reductions needed to attain MDG5 and most births and maternal deaths occur at home without the attention of the health service.
